# Supplementary material for: Translational fidelity and growth of Arabidopsis require stress-sensitive diphthamide biosynthesis
Source: Nat Commun. 2022 Jul 11;13:4009. doi: 10.1038/s41467-022-31712-7 (PMC9273596; doi:10.1038/s41467-022-31712-7)
Supplement: Supplementary file 1 — Supplementary Information [file 41467_2022_31712_MOESM1_ESM.pdf]

Supplementary Information

Translational fidelity and growth of Arabidopsis require stress-sensitive diphthamide biosynthesis

Hongliang Zhang, Julia Quintana, Koray Ütkür, Lorenz Adrian, Harmen Hawer, Klaus Mayer, Xiaodi Gong, Leonardo Castanedo, Anna Schulten, Nadežda Janina, Marcus Peters, Markus Wirtz, Ulrich Brinkmann, Raffael Schaffrath and Ute Krämer

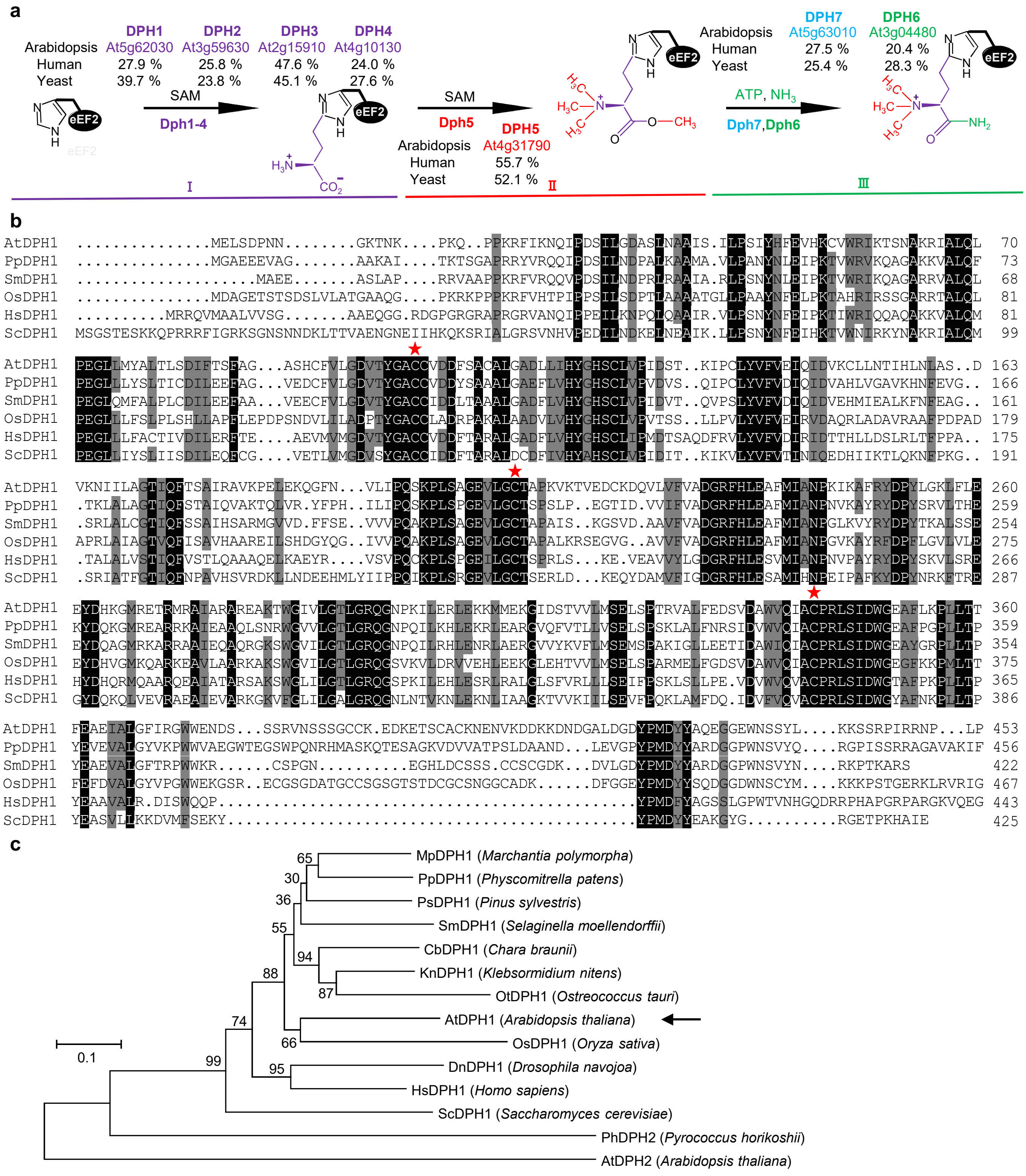

Supplementary Fig. 1 Diphthamide biosynthesis proteins and identification of homologues in Arabidopsis. a, Scheme of biosynthetic pathway of the diphthamide modification on eEF2, organized into steps I to III, listing the contributing human and yeast proteins with their putative homologues in Arabidopsis and percent identical amino acids in a sequence alignment. **b**, Sequence alignment of DPH1 homologues. Three conserved Cys residues thought to be critical for Fe-S cluster binding are marked by red asterisks (above). Poorly aligned C-terminal residues are not shown (At 4, Pp 11, Sm 0, Os 11, Hs 24, Sc 0 amino acids) **c**, Neighbor-joining tree of DPH1 homologues. Numbers at the branching points specify support (%) from bootstrap analysis (1,000 iterations). Scale bar indicates 0.1 substitutions per amino acid position (sum of branch lengths 4.61). AtDPH2 is included to represent eukaryotic DPH2 proteins.

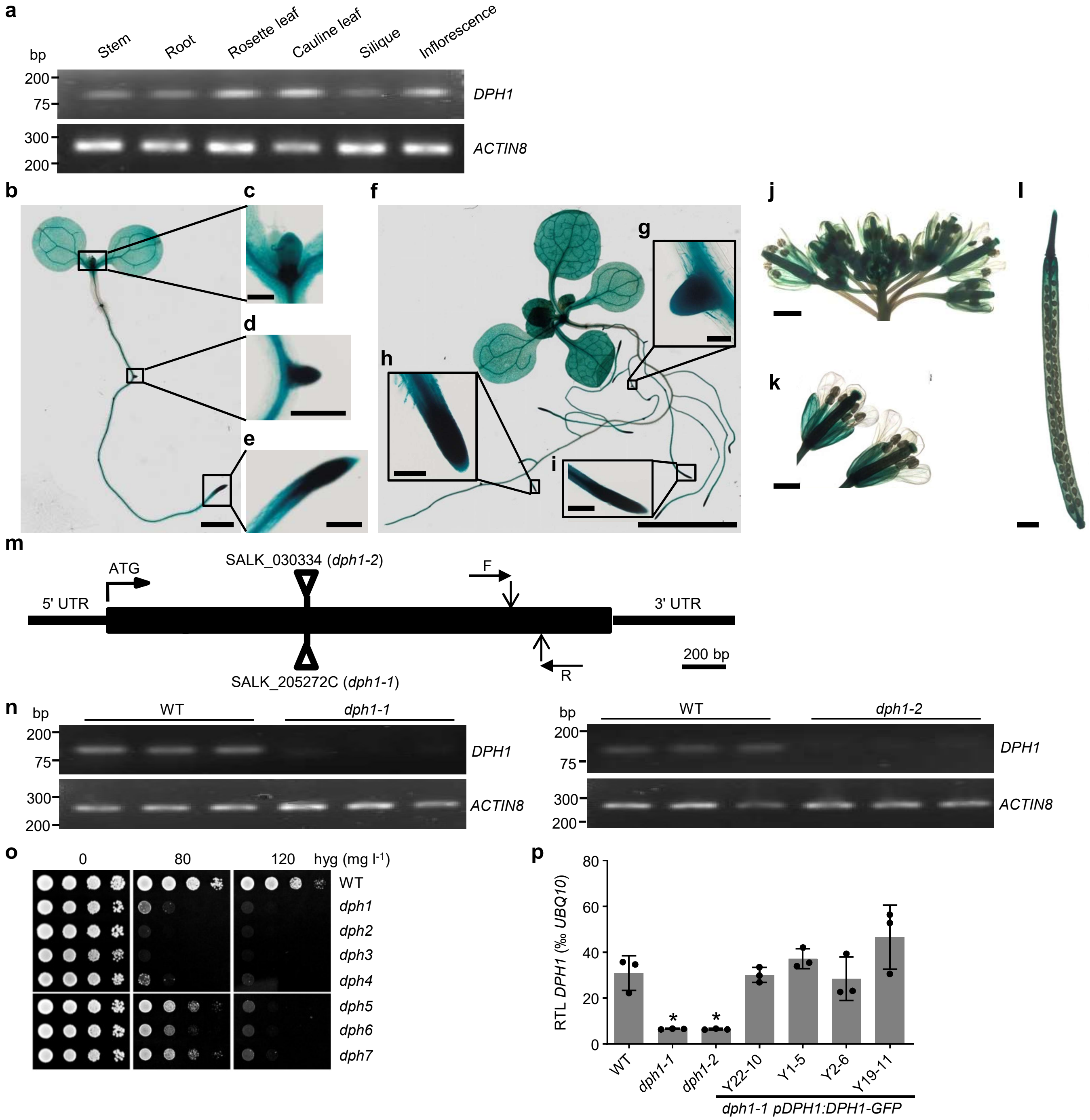

Supplementary Fig. 2 *DPH1* expression and identification of Arabidopsis *dph1* mutants. a, Detection of *DPH1* transcript (30 cycles) in various tissues of 4-week-old soil-grown Arabidopsis (Col-0) wild-type plants (WT) by RT-PCR, alongside *ACTIN8* as a constitutively expressed control gene (28 cycles). Image shown is from one experiment and representative of three independent experiments. **b**-**l**, Histochemical β-glucuronidase (GUS) activity staining in *pDPH1:GUS* plants. One-week-old seedling (**b**), with enlargened shoot apex (**c**) and lateral (**d**) and primary (**e**) root tips. Two-week-old seedling (**f**), with enlargened root tips (**g**-**i**). Shoot apex (reproductive stage, **j**), flowers (**k**), developing silique (**l**). Seedlings were grown on 0.5x MS medium (**b**-**i**), and other tissues were harvested from 7-week-old soil-grown plants (**j**-**l**). **m**, Schematic representation of T-DNA insertions at the *DPH1* locus in *dph1-1* and *dph1-2* mutants. The positions of 5' ends of primers used for RT-PCR (**a**,**n**) and RT-qPCR (**p**) are indicated (F: forward primer; R: reverse primer). **n**, Detection of *DPH1* transcript in leaves of 4-week-old WT, *dph1-1* and *dph1-2* plants by RT-PCR (see **a**). Each of the three lanes per genotype corresponds to an independent experiment. **o**, Yeast hygromycin sensitivity assay. Ten-fold serial dilutions of the wild-type and each *dph* mutant yeast strain were spotted onto YPD media containing hygromycin B (hyg) as indicated, followed by incubation at 30°C for 2 d. **p**, RT-qPCR quantification of *DPH1* transcript levels in shoots of 5-week-old soil-grown WT plants, *dph1* mutants, and *dph1-1 pDPH1:DPH1-GFP* complemented lines. Data are mean ± s.d., $n = 3$ biologically independent plants per genotype. Significant differences from WT: *, $P < 0.05$, one-way ANOVA with Tukey's test. Scale bars: 1 mm (**b**,**j**-**l**); 0.2 mm (**c**-**e**,**i**); 5 mm (**f**); 50 µm (**g**); 100 µm (**h**).

a

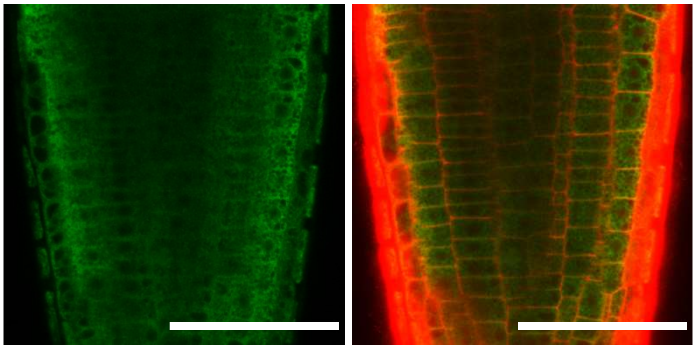

b

```
AtEF2    MVKFTADELRRIMDYKHNIRNMSVIAHVDHGKSTLTDSLVAAAGIIAQEVAGDVRMTDTRADEAERGITIKSTGISLYYEMTDESLKSFTGARDGNEYLI    100
HsEF2    MVNFTVDQIRAIMDKKANIRNMSVIAHVDHGKSTLTDSLVCKAGIIASARAGETRFTDTRKDEQERCITIKSTAISLFYELSENDLNFIKQSKDGAGFLI    100
ScEF2    MVAFTVDQMRSLMDKVTNVRNMSVIAHVDHGKSTLTDSLVQRAGIISAAKAGEARFTDTRKDEQERGITIKSTAISLYSEMSDEDVKEIKQKTDGNSFLI    100

AtEF2    NLIDSPGHVDFSSEVTAALRITDGALVVVDCIEGVCVQTETVLRQALGERIRPVLTVNKMDRCFLELQVDGEEAYQTFSRVIENANVIMATY...EDPLL    197
HsEF2    NLIDSPGHVDFSSEVTAALRVTDGALVVVDCVSGVCVQTETVLRQAIAERIKPVLMMNKMDRALLELQLEPEELYQTFQRIVENVNVIISTYGEGESGPM    200
ScEF2    NLIDSPGHVDFSSEVTAALRVTDGALVVVDTIEGVCVQTETVLRQALGERIKPVVVINKVDRALLELQVSKEDLYQTFARTVESVNVIVSTY...ADEVL    197

AtEF2    GDVQVYPEKGTVAFSAGLHGWAFTLTNFAKMYASKFGVVES.............KMMERLWGENFFDPATRKWSGKNTGSPTCK...RGFVQFCYEPIKQ    281
HsEF2    GNIMIDPVLGTVGFGSGLHGWAFTLKQFAEMYVAKFAAKGEGQLGPAERAKKVEDMMKKLWGDRYFDPANGKFS.KSATSPEGKKLPRTFCQLILDPIFK    299
ScEF2    GDVQVYPARGTVAFGSGLHGWAFTIRQFATRYAKKFGVDKA.............KMMDRLWGDSFFNPKTKKWT.NKDTDAEGKPLERAFNMFILDPIFR    283

AtEF2    IIATCMNDQKDKLWPMLAKLGVSMKNDEKELMGKPLMKRVMQTWLPASTALLEMMIFHLPSPHTAQRYRVENLYEGPLDDQYANAIRNCDPNGPLMLYVS    381
HsEF2    VFDAIMNFKKEETAKLIEKLDIKLDSEDKDKEGKPLLKAVMRRWLPAGDALLQMITIHLPSPVTAQKYRCELLYEGPPDDEAAMGIKSCDPKGPLMMYIS    399
ScEF2    LFTAIMNFKKDEIPVLLEKLEIVLKGDEKDLEGKALLKVVMRKFLPAADALLEMIVLHLPSPVTAQAYRAEQLYEGPADDANCIAIKNCDPKADLMLYVS    383

AtEF2    KMIPASDKGRFFAFGRVFAGKVSTGMKVRIMGPNYIPGEKKDLYTKSVQRTVIWMGKRQETVEDVPCGNTVAMVGLDQFITKNATLTNEKEVDAHPIRAM    481
HsEF2    KMVPTSDKGRFYAFGRVFSGLVSTGLKVRIMGPNYTPGKKEDLYLKPIQRTILMMGRYVEPIEDVPCGNIVGLVGVDQFLVKTGTITT..FEHAHNMRVM    497
ScEF2    KMVPTSDKGRFYAFGRVFAGTVKSGQKVRIQGPNYVPGKKDDLFIKAIQRVVLMMGRFVEPIDDCPAGNIIGLVGIDQFLLKTGTLTT..SETAHNMKVM    481

AtEF2    KFSVSPVVRVAVQCKVASDLPKLVEGLKRLAKSDPMVVCTMEESGEHIVAGAGELHLEICLKDLQDDFMGGAEIIKSDPVVSFRETVCDRSTRTVMSKSP    581
HsEF2    KFSVSPVVRVAVEAKNPADLPKLVEGLKRLAKSDPMVQCIIEESGEHIIAGAGELHLEICLKDLEEDHAC.IPIKKSDPVVSYRETVSEESNVLCLSKSP    596
ScEF2    KFSVSPVVQVAVEVKNANDLPKLVEGLKRLSKSDPCVLTYMSESGEHIVAGTGELHLEICLQDLEHDHAG.VPLKISPPVVAYRETVESESSQTALSKSP    580

AtEF2    NKHNRLYMEARPMEEGLAEAIDDGRIGPRDDPKIRSKILAEEFGWDKDLAKKIWAFGPETTGPNMVVDMCKGVQYLNEIKDSVVAGFQWASKEGPLAEEN    681
HsEF2    NKHNRLYMKARPFPDGLAEDIDKGEVSARQELKQRARYLAEKYEWDVAEARKIWCFGPDGTGPNILTDITKGVQYLNEIKDSVVAGFQWATKEGALCEEN    696
ScEF2    NKHNRIYLKAEPIDEEVSLAIENGIINPRDDFKARARIMADDYGWDVTDARKIWCFGPDGNGPNLVIDQTKAVQYLHEIKDSVVAAFQWATKEGPIFGEE    680

AtEF2    MRGICFEVCDVVLHSDAIHRGGGQVIPTARRVIYASQITAKPRLLEPVYMVEIQAPEGALGGIYSVLNQKRGHVFEEMQRPGTPLYNIKAYLPVVESFGF    781
HsEF2    MRGVRFDVHDVTLHADAIHRGGGQIIPTARRCLYASVLTAQPRLMEPIYLVEIQCPEQVVGGIYGVLNRKRGHVFEESQVAGTPMFVVKAYLPVNESFGF    796
ScEF2    MRSVRVNILDVTLHADAIHRGGGQIIPTMRRATYAGFLLADPKIQEPVFLVEIQCPEQAVGGIYSVLNKKRGQVVSEEQRPGTPLFTVKAYLPVNESFGF    780

AtEF2    SSQLRAATSGQAFPQCVFDHWEMMSSDPLEPGTQASVLVADIRKRKGLKEAMTPLSEFEDK    842
HsEF2    TADLRSNTGGQAFPQCVFDHWQILPGDPFDNSSRPSQVVAETRKRKGLKEGIPALDNFLDK    857
ScEF2    TGELRQATGGQAFPQMVFDHWSTLGSDPLDPTSKAGEIVLAARKRHGMKEEVPGWQEYYDK    841
```

Supplementary Fig. 3 Data supporting sub-cellular localization of AtDPH1, and sequence alignment of eEF2 proteins. a, Representative confocal laser scanning microscopic image of a root tip of an 8-d-old *dph1-1 pDPH1:DPH1-GFP* (line Y22-10) stained with propidium iodide (PI, red; see **Fig. 2a**). Scale bars, 50 µm. **b**, Sequence alignment of eEF2 of Arabidopsis, human and yeast. The amino acids corresponding to the HsEF2 peptide used to generate the 10G8 antibody specific for diphthamide-unmodified eEF2 are boxed in red. The conserved His residue known to carry the diphthamide modification in yeast and human is marked by a blue triangle (Yeast: His699, Human: His715, Arabidopsis: His700).

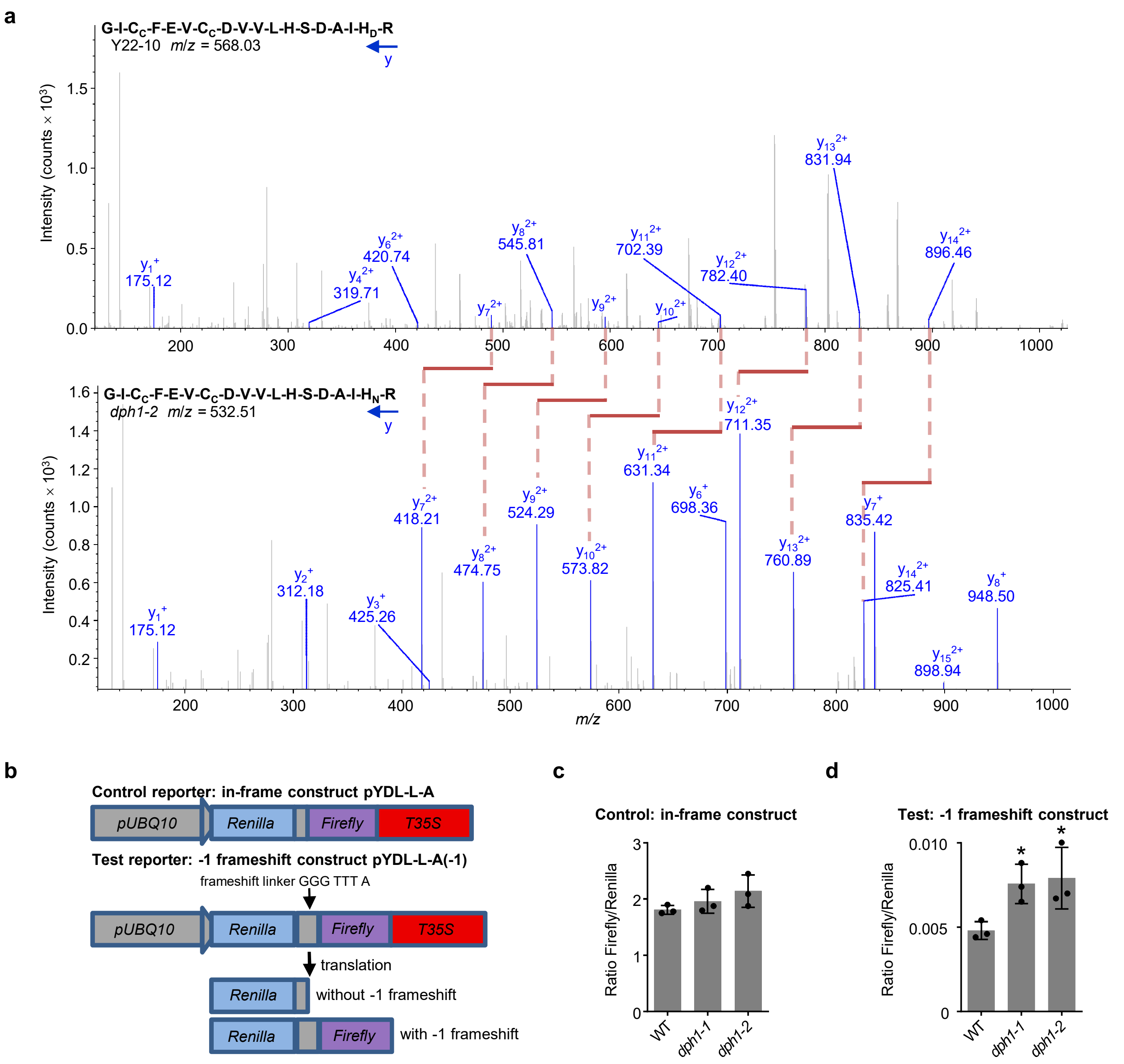

Supplementary Fig. 4 Arabidopsis *dph1* mutants lack diphthamide modification of eEF2 and exhibit enhanced rates of translational -1 frameshifting. a, MS/MS spectra of eEF2 peptide 684-GICFEVCDVVLHSDAIHR-701 from *dph1-1 pDPH1:DPH1-GFP* (line Y22-10, top) and the *dph1-2* mutant (bottom), with diphthamide ($_D$) or without diphthamide ($_N$) modification on H700 respectively ($_C$: carbamidomethylation). The selected monoisotopic precursor m/z (z = +4) is given in each diagram; all detected y fragment ions are shown in blue. Red/pink lines visualize the consistent m/z difference of 71.055 between equivalent y_n^{2+} ions of the two genotypes (see **Fig. 2c**). **b**, Schematic representation of the reporter system used for quantifying ribosomal -1 frameshifting error (see **Fig. 2d**). The system consists of pYDL-L-A as a control reporter and pYDL-L-A(-1) as a test reporter. Both reporters encode a chimeric fusion protein of renilla luciferase and firefly luciferase, separated by a linker. For the control reporter, the firefly luciferase is in frame with renilla luciferase. For the test reporter, the firefly luciferase is in +1 frame relative to renilla luciferase - a fusion protein is only produced if -1 frameshifting occurs during the translation of the linker sequence. **c**-**d**, Ratios of firefly to renilla luciferase activities in Arabidopsis mesophyll protoplasts. Mesophyll protoplasts isolated from the wild type (WT) and *dph1* mutants were transfected with pYDL-L-A (control, **c**) and pYDL-L-A(-1) (test, **d**), respectively (see **Fig. 2d**). Data are mean ± s.d. (n = 3 independently transformed replicate aliquots of protoplasts). Significant differences from WT: *, P < 0.05, one-way ANOVA with Tukey's test.

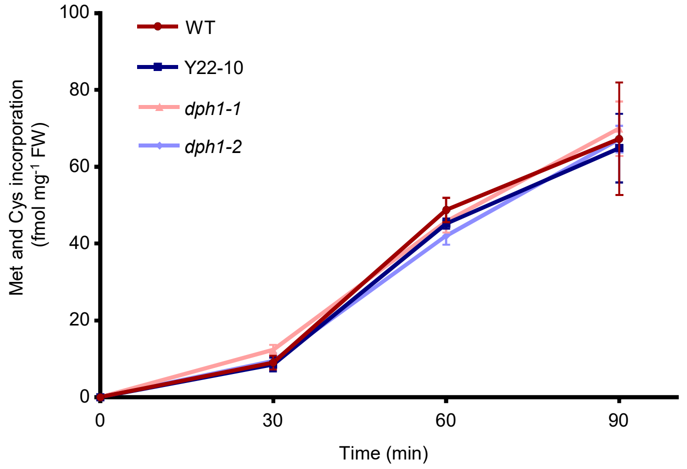

Supplementary Fig. 5 Global protein biosynthesis rates. Shown is the amount of the amino acids methionine and cysteine incorporated into newly synthesized proteins in leaf disks of 5-week-old soil-grown plants over the labeling period with ^{35}S-radiolabeled amino acids. WT: wild type; Y22-10: *dph1-1 pDPH1:DPH1-GFP* complemented line Y22-10; FW: fresh biomass. Data are mean ± s.d., *n* = 3 plants, with four leaf disks per plant.

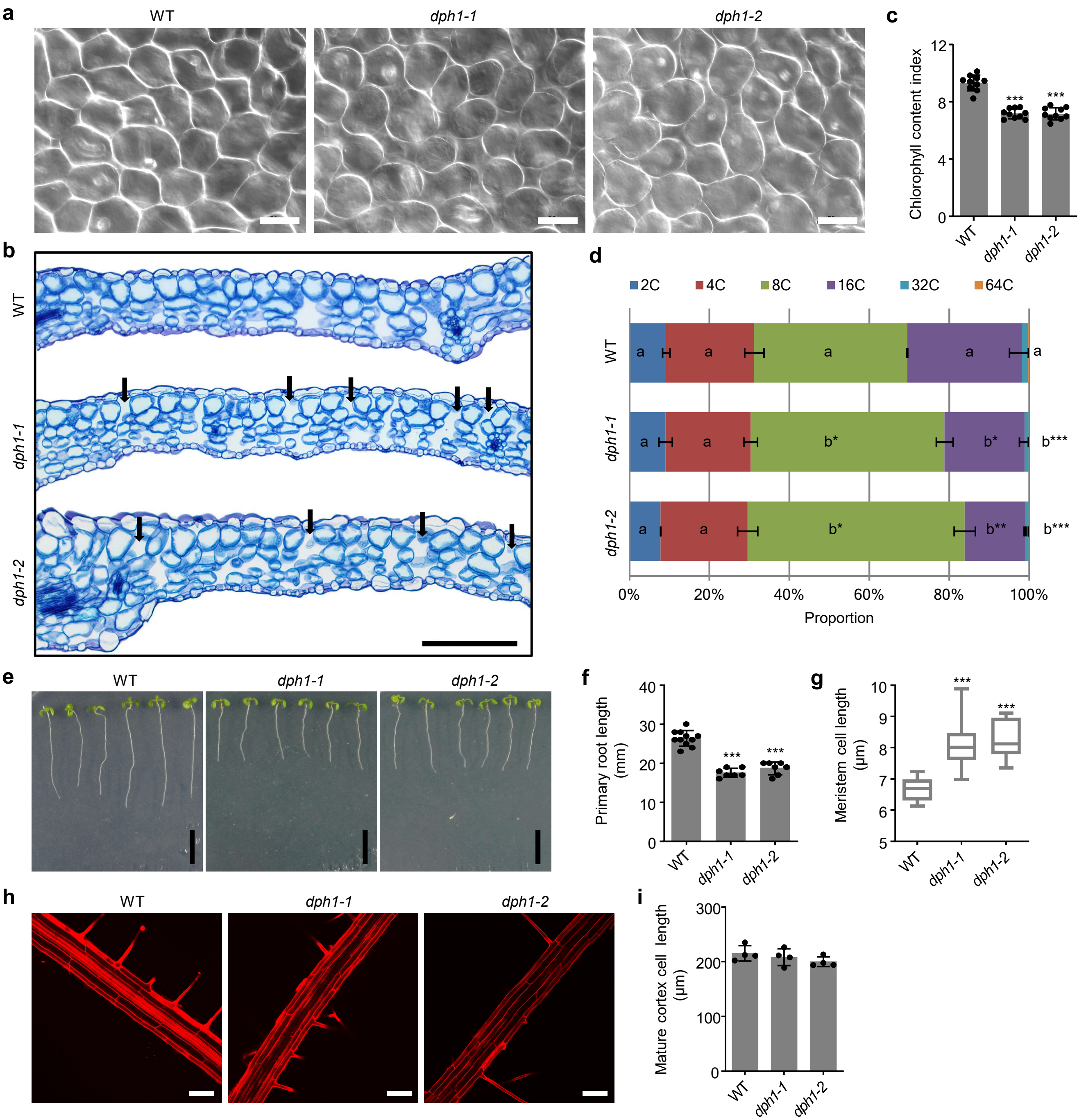

Supplementary Fig. 6 Raw and supporting data on the reduced growth of *dph1* mutants. a-c, Photographs showing palisade cells (**a**), transverse sections of the central region of leaf blades stained with toluidine blue (**b**), and bargraph of chlorophyll content index (*n* = 10 replicate plants per genotype, **c**), for the 6th oldest leaves shown in **Fig. 3b**. Scale bars, 50 µm (**a**). Arrows indicate the expanded intercellular spaces between the palisade cells of *dph1* mutants; scale bar, 200 µm (**b**). (**d**) Distribution of nuclear ploidy in the first pair of true leaves of 3-week-old soil-grown wild-type (WT) and *dph1* mutants (*n* = 3 independent experiments). Different characters in segments sharing the same color indicate statistically significant differences. **e**, Photographs of 9-d-old WT and *dph1* seedlings cultivated on 0.5x MS medium. Scale bars, 10 mm. **f**, Primary root length of WT and *dph1* seedlings shown in (**e**), with *n* = 11 seedlings for WT, *n* = 7 seedlings for *dph1* mutants. **g**, Meristem cell length of the seedlings shown in **Fig. 3h**, with median (center), 1st and 3rd quartile (box), minimum and maximum (whiskers), *n* = 12 replicate seedlings per genotype. **h**, Confocal laser scanning microscopic images of differentiation zone of representative PI-stained roots of WT and *dph1* seedlings shown in (**e**). Scale bars, 100 µm. **i**, Cortex cell length in root differentiation zone of the seedlings shown in (**h**). Cell size was measured with ImageJ software (*n* = 52 for WT, *n* = 48 for *dph1-1*, and *n* = 64 for *dph1-2* from a total of 4 seedlings per genotype). Data are mean ± s.d. (**c**,**d**,**f**,**i**), or shown as box-and-whisker plot (**g**); significant differences from WT: *, $P < 0.05$, **, $P < 0.01$, ***, $P < 0.001$, one-way ANOVA with Tukey's test (**c**,**d**,**g**,**i**) or Scheffé test (**f**).

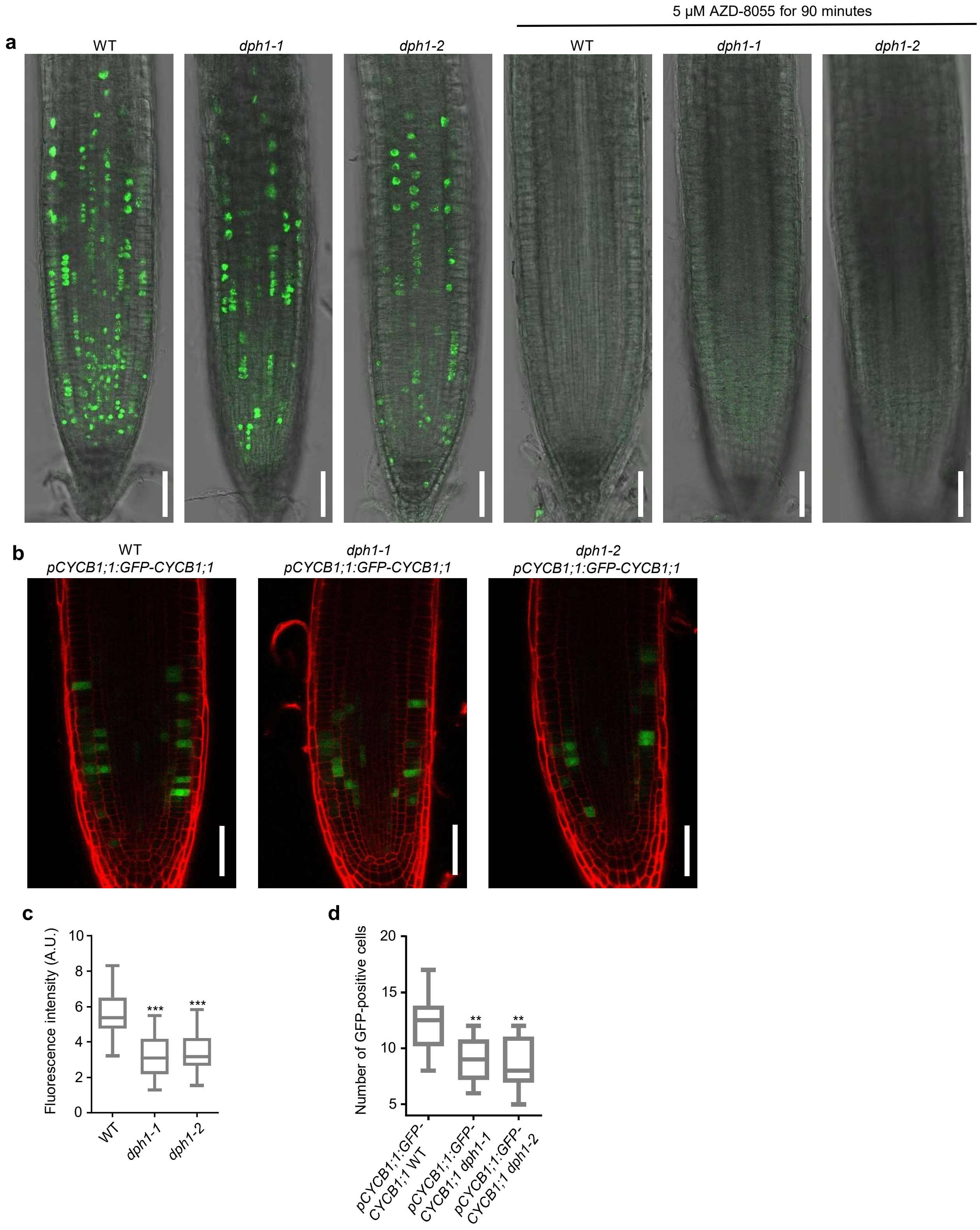

Supplementary Fig. 7 Decreased cell proliferation in root tips of *dph1* mutants. a, Confocal microscopic images of EdU (5-ethynyl-2′-deoxyuridine)-stained root tips of intact seedlings. DNA that was newly synthesized during a 30-min period of labeling with the thymidine analogue EdU is marked by a green fluorescent signal. **b**, Confocal microscopic images of root tips of seedlings carrying a *pCYCB1;1:GFP-CYCB1;1* transgene in the wild-type (left) and *dph1* mutant backgrounds (middle and right). Cells in the late G2 phase and in the M phase of the cell cycle are marked by a green fluorescent signal. Propidium iodide (red fluorescence) was used as a counterstain. **c**, Quantification of the fluorescent signal per root tip (frame), with representative images shown in (**a**). **d**, Quantification of the number of cells showing GFP fluorescence, with representative images shown in (**b**). Shown are median (center line), 1st and 3rd quartile (box), minimum and maximum (whiskers)(**c**,**d**), of $n = 18$ (**a**, **c**) and $n = 12$ (**b**,**d**) replicate seedlings per genotype; significant differences from wild type (WT): **, $P < 0.01$, ***, $P < 0.001$, one-way ANOVA with Tukey's test (**c**,**d**). Seedlings were cultivated on 0.5x MS medium for 8 d. All images were taken in the central confocal plane of the root tip using identical settings; scale bars, 50 μm (**a**,**b**). A.U., arbitrary unit.

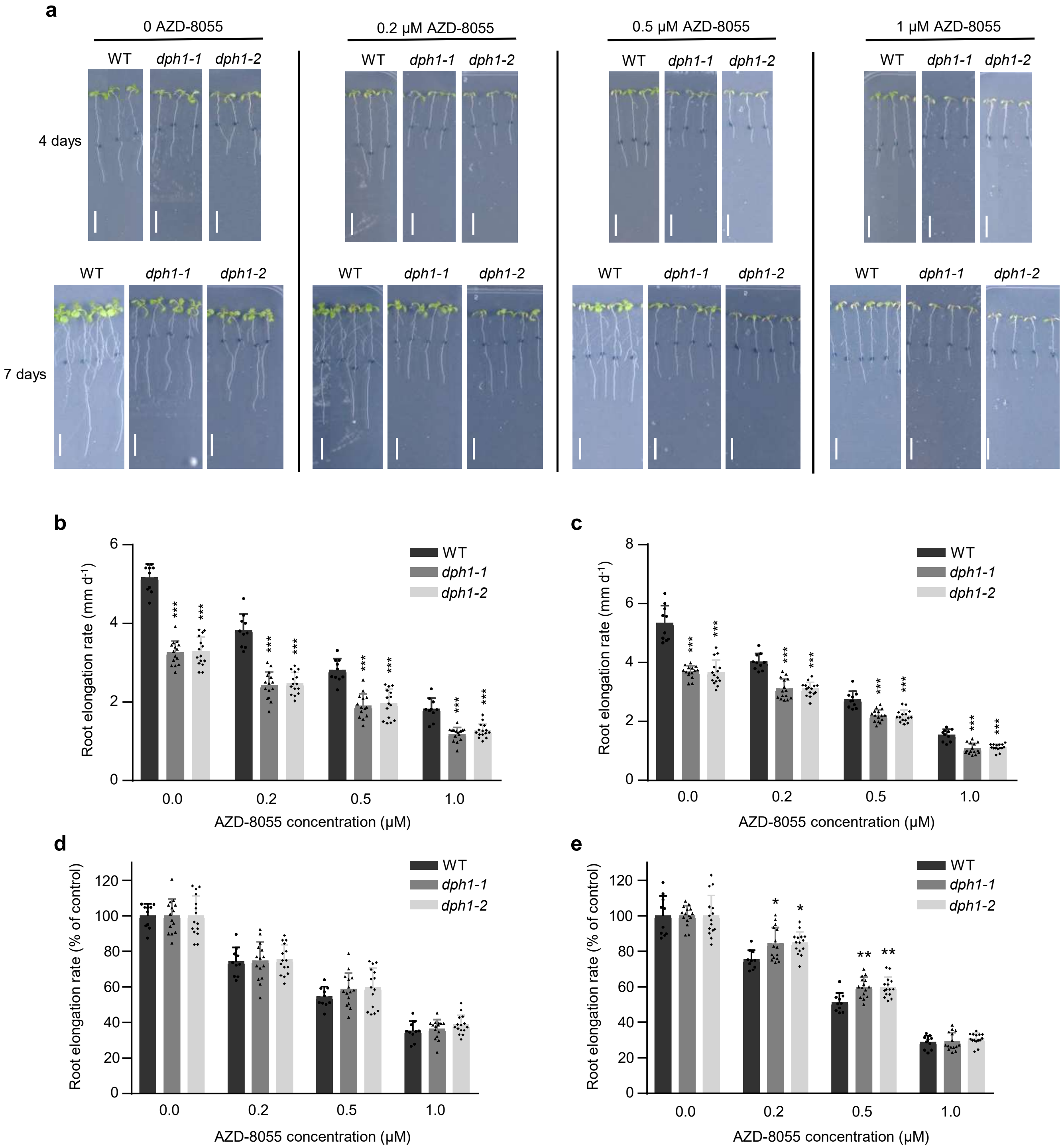

Supplementary Fig. 8 Sensitivity of root elongation to the TOR inhibitor AZD-8055. a, Representative images of wild type (WT) and *dph1* mutant seedlings upon exposure to a range of AZD-8055 concentrations for 4 d (top row) and 7 d (second row). **b**,**c**, Root elongation rates after 4 d (**b**) and 7 d (**c**) of AZD-8055 treatment, as shown in (**a**). Data are mean ± s.d., *n* = 10 and 15 seedlings for WT and *dph1* mutants, respectively. **d**,**e**, Data as in (**b**, **c**) shown normalized to the mean of same genotype under the control condition. Seedlings were pre-cultivated on agar-solidified 0.5x MS medium for 7 d and subsequently transferred onto fresh medium without (control) or with various concentrations of AZD-8055 as indicated. Root lengths were measured in photographic images using ImageJ, and scale bars are 10 mm (**a**). Significant differences from WT: *, $P < 0.05$, **, $P < 0.01$ ***, $P < 0.001$, one-way ANOVA with Scheffé test (**b**-**e**).

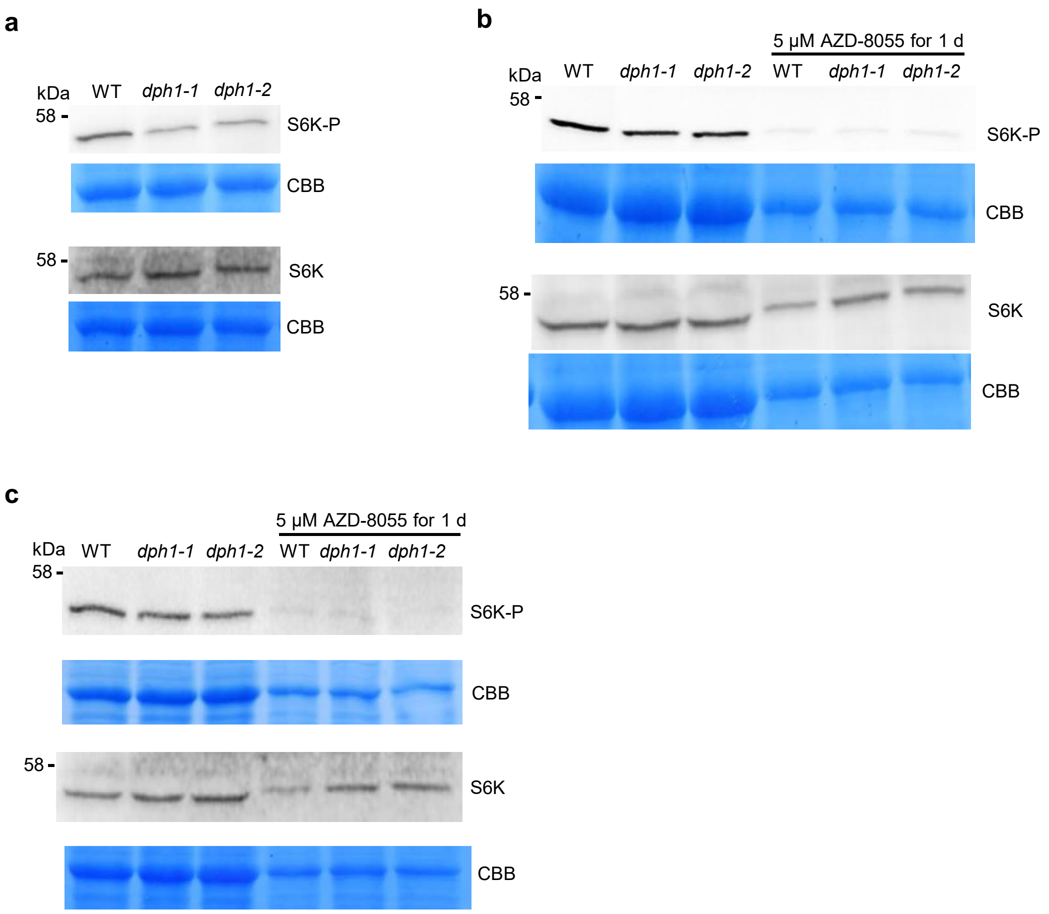

Supplementary Fig. 9 Reduced TOR activity in *dph1* mutants. a-**c**, Additional immunoblots reflecting TOR activity according to the levels of S6K-P and S6K in 14-day-old wild-type (WT) and *dph1* mutant seedlings (employed to calculate mean values shown in **Fig. 3m**). Total protein extracts were resolved by SDS-PAGE, blotted and probed with antibodies against S6K-P and S6K, respectively. Each panel is from one independent experiment (**a**-**c**). Seedlings were cultivated in liquid 0.5x MS salts supplemented with 0.5% (w/v) sucrose. Seedlings treated with 5 μM TOR inhibitor AZD-8055 for 1 day were used as controls in (**b**) and (**c**).

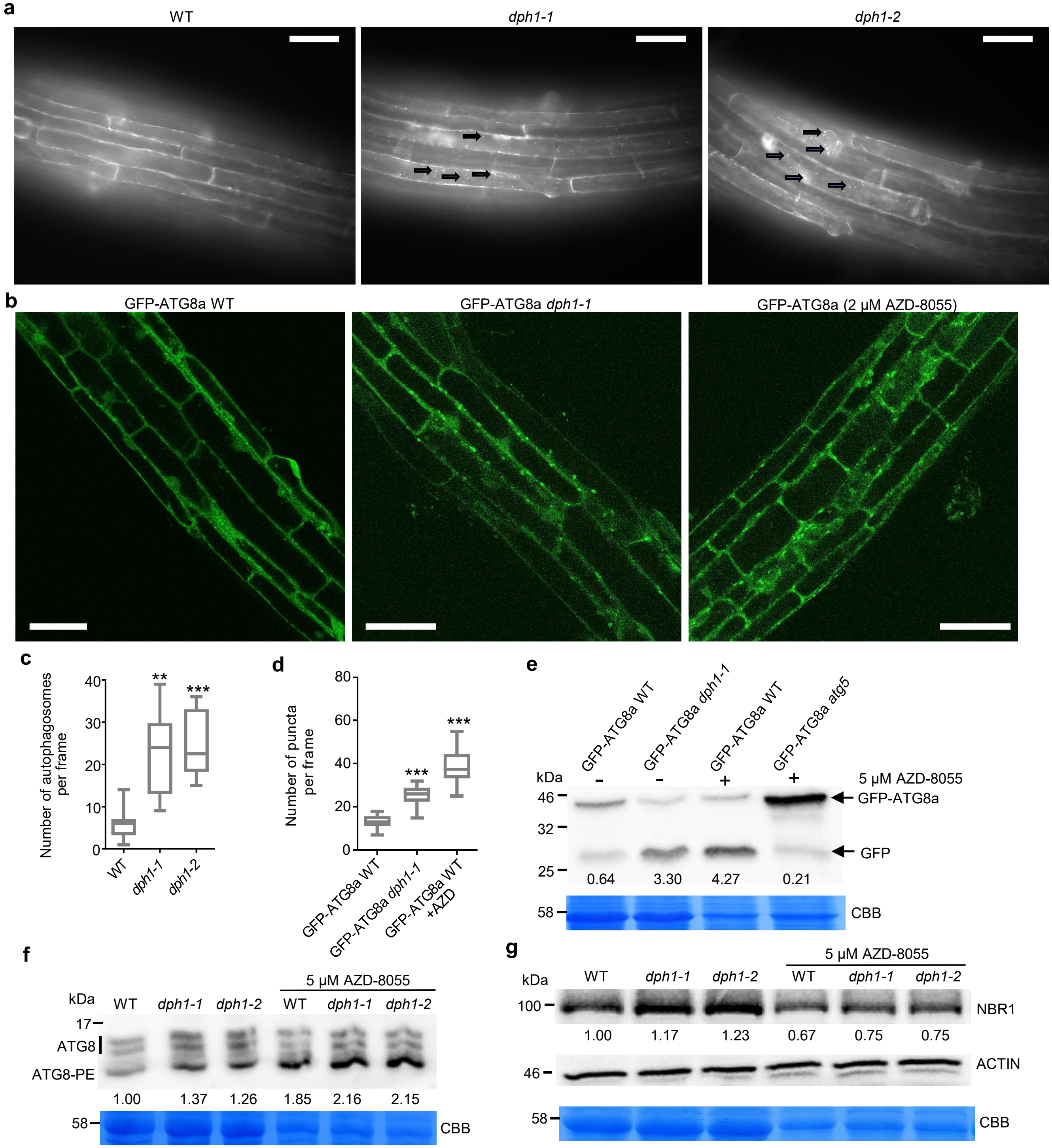

Supplementary Fig. 10 Autophagy is activated in *dph1* mutants. a, Fluorescent microscopic images of autophagosomes labeled with the fluorescent dye monodansylcadaverine (MDC). Shown are the root elongation zones of 7-d-old wild-type (WT) and *dph1* mutant seedlings (see Methods). Arrows indicate examples of autophagosomes, structures formed during autophagy induction. **b**, Confocal microscopic images of GFP-ATG8a *punctae* in roots of 8-day-old seedlings (see Methods). **c**-**d**, Number per frame of autophagosomes (**c**) as shown in (**a**), and of autophagosome-related *punctae* (**d**) as shown in (**b**), median (center), 1st and 3rd quartile (box), minimum and maximum (whiskers), n = 16 (WT) and n = 8 (*dph1*) (**c**), and n = 16 (**d**) biologically independent seedlings per genotype, with one frame per seedling. **e**, Autophagy induction in the *dph1-1* mutant as diagnosed using the GFP-ATG8a cleavage assay. Immunoblot shows GFP-ATG8a and released free GFP for the genotypes as indicated. *atg5* mutants accumulate GFP-ATG8a under AZD-8055 treatment, reflecting impaired autophagic flux. **f**-**g**, Immunoblot analysis of autophagy-related ATG8 (**f**) with the possible position of the lipidated ATG8-phospatidyl-ethanolamine form marked as ATG8-PE, and NBR1 (**g**). Total protein extracts were resolved by SDS-PAGE, and blots were probed with anti-GFP (**e**), anti-ATG8 (**f**), or anti-NBR1 (**g**) antibodies, as well as probed with an anti-ACTIN antibody or stained with Coomassie Brilliant Blue (CBB) as loading controls. Numbers below the blot images are the ratios of GFP to GFP-ATG8a band intensities for each lane (**e**), and ATG8-PE (**f**) or NBR1 (**g**) band intensities relative to WT. Seedlings were pre-cultivated in agar-solidified 0.5x MS medium for 7 d, then cultivated in liquid 0.5x MS medium containing 0.5% (w/v) sucrose for 6 d, followed by addition of the TOR kinase inhibitor AZD-8055 for 24 h (**e**,**f**,**g**). Significant differences from the respective WT: **, $P < 0.01$, ***, $P < 0.001$, one-way ANOVA with Games-Howell test (**c**,**d**). Scale bars, 50 µm (**a**,**b**).

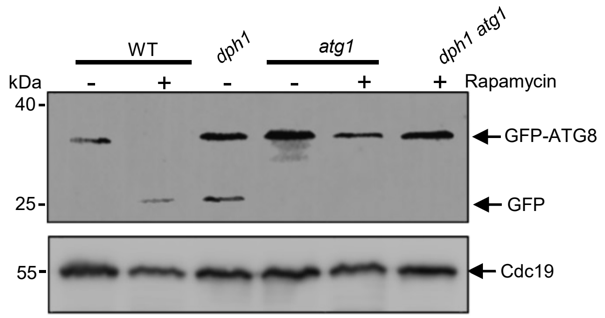

Supplementary Fig. 11 Autophagy is induced in the *dph1* mutant of *Saccharomyces cerevisiae*. Immunoblots show GFP-ATG8 or released free GFP (upper panel) for the wild type (WT) and the *dph1* mutant. Rapamycin (+) inhibition of TOR activity in WT is shown as a positive control for autophagy induction, and autophagy-defective *atg1* single and *dph1 atg1* double mutants are shown as negative autophagy controls. Pyruvate kinase (Cdc19, lower panel) is shown as a loading control. Data shown are from one experiment and representative of a total of four independent experiments.

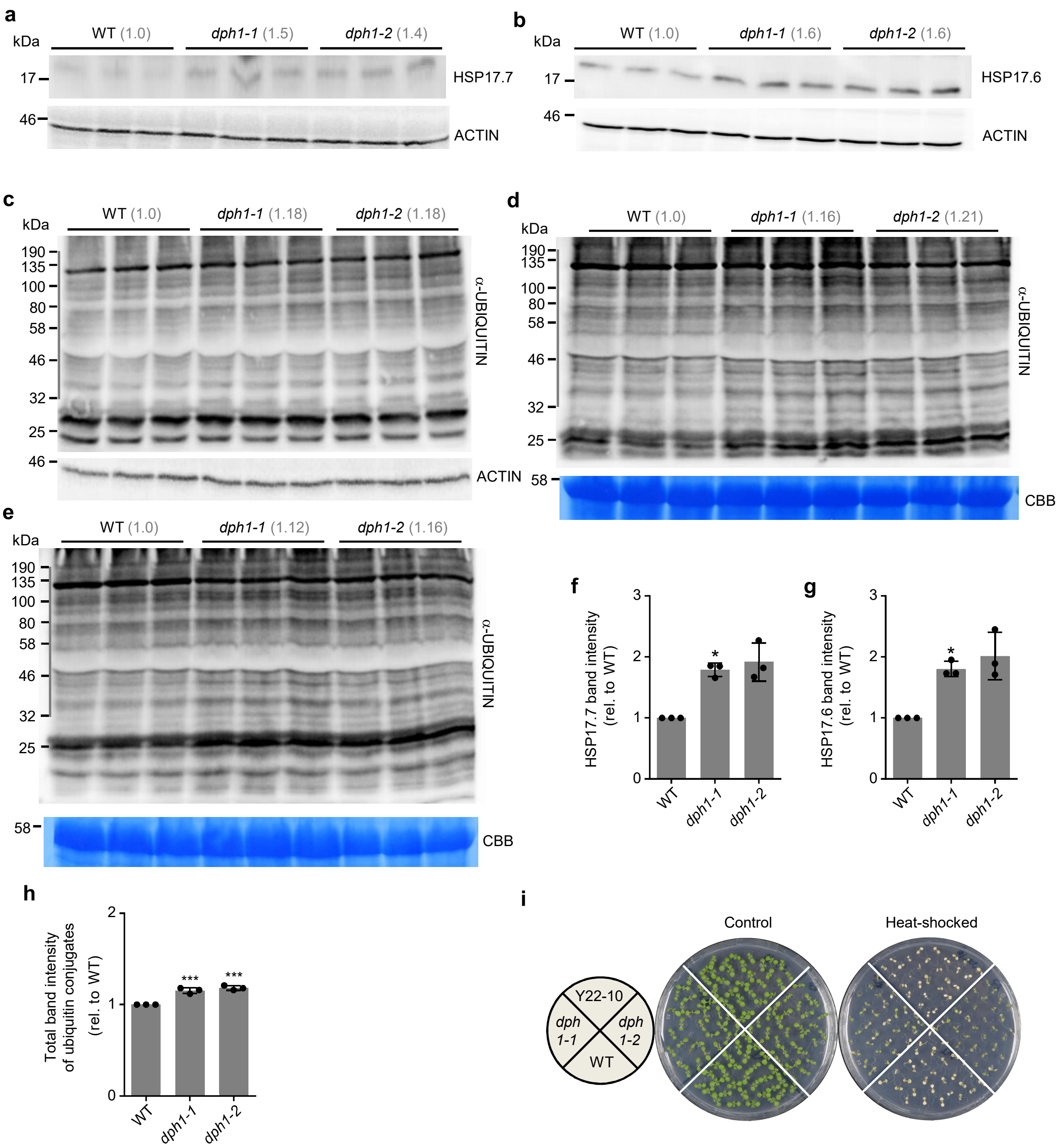

Supplementary Fig. 12 Enhanced protein aggregation in *dph1* mutants. a-**e**, Immunoblot detection of HSP 17.7 (**a**), HSP 17.6 (**b**), and ubiquitin conjugates (**c-e**) in shoot tissues of four-week-old plants of the indicated genotypes grown in soil. Each of the three lanes per genotype corresponds to one independent experiment. Shown are three replicate immunoblots (**c-e**) conducted with protein extracts of different aliquots of the same tissue homogenates, with the size range used for quantification marked by a grey vertical line on the left of the blot image. For all immunoblots, total protein extracts were resolved by SDS-PAGE and probed with the corresponding primary antibodies. Numbers in parentheses are band intensities relative to those in wild type (WT), all normalized to the respective loading control (means of n = 4 technical replicate quantifications) . **f-h**, Quantification of band intensities of HSP17.7 (**f**), HSP17.6 (**g**), and ubiquitin conjugates (**h**) as shown in (**a**), (**b**) and (**c-e**), respectively (mean ± s.d., n = 3 independent experiments for **f** and **g**; n = 3 replicate immunoblots for **h**). Significant differences from WT: *, $P < 0.05$, **, $P < 0.01$, one-way ANOVA with Games-Howell test (**f**,**g**) or Tukey's test (**h**). **i**, Heat stress tolerance. One-week-old seedlings of WT, *dph1* mutants, and *dph1-1 pDPH1:DPH1-GFP* (line Y22-10) grown on 0.5x MS medium were exposed to 50°C for 50 minutes. Photos were taken after recovery for 3 days.

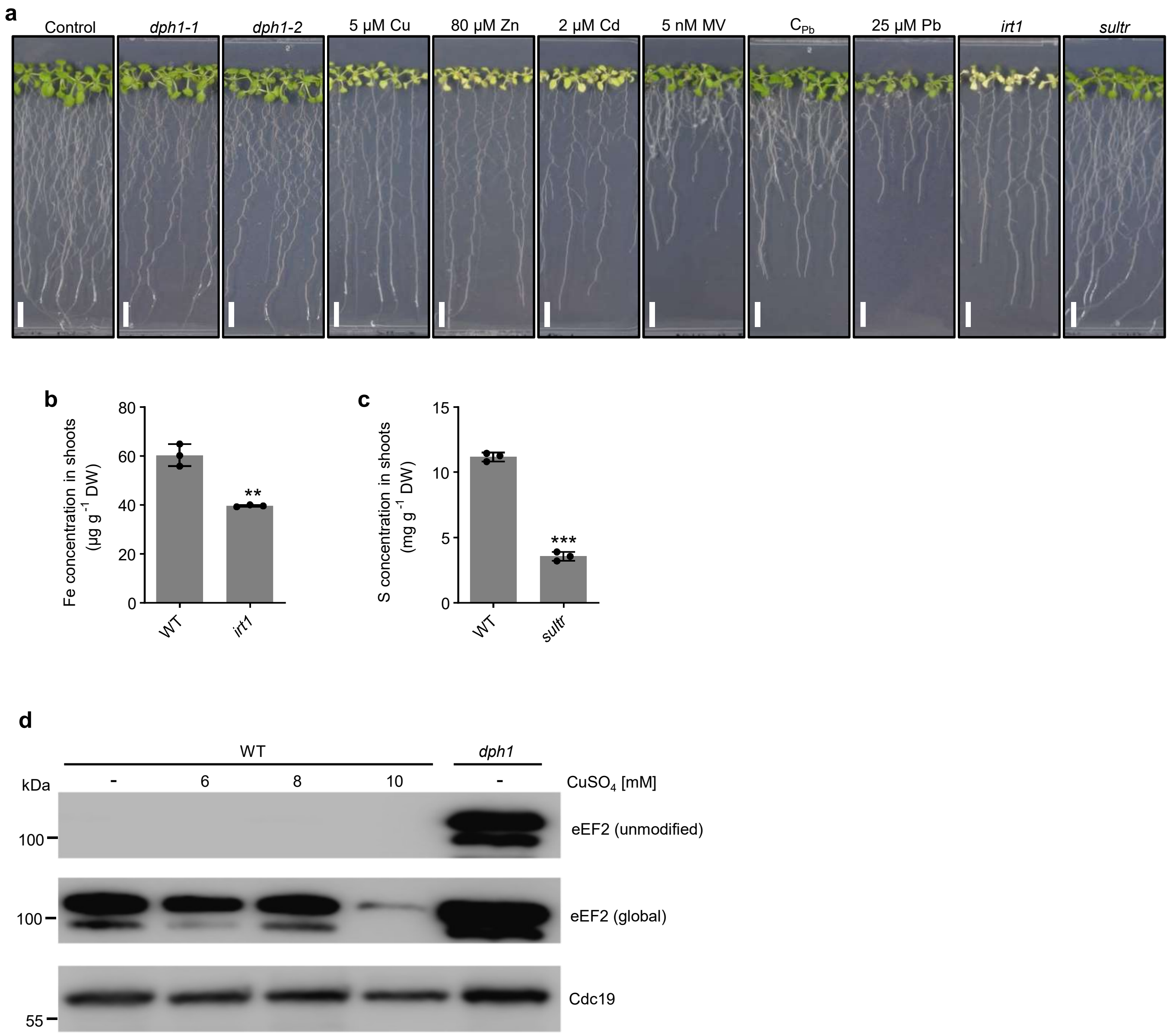

Supplementary Fig. 13 Data supporting the accumulation of diphthamide-unmodified eEF2 protein under abiotic stress. **a**, Photographs of 18-d-old iron uptake-defective *irt1,* and sulfate uptake-defective *sultr1;1 sultr1;2 (sultr)* mutants*,* as well as wild-type (WT), *dph1-1* and *dph1-2* seedlings grown in modified Hoagland medium (Control), or WT in modified Hoagland medium supplemented with 5 µM Cu, 80 µM Zn, 2 µM Cd, 5 nM MV, or 25 µM Pb by comparison to a specific Pb-free control medium (C_{Pb})(see **Fig. 5a**). Scale bars, 10 mm. **b**-**c**, Shoot Fe (**b**) and shoot total S (**c**) concentrations in a subset of seedlings shown in (**a**). Shown are mean ± s.d., n = 3 biologically independent samples, with each sample corresponding pooled tissues from 17 seedlings cultivated per petri plate, **, $P < 0.01$, ***, $P < 0.001$, compared to WT, according to two-tailed Student's *t*-test compared with WT (**b**,**c**). **d**, Immunoblot detection of unmodified and global eEF2 protein in total protein extracts from WT and the *dph1* mutant of *Saccharomyces cerevisiae* cultivated in liquid YPD medium without or with addition of $CuSO_4$. Yeast cultures were harvested at an OD_{600} of 1.

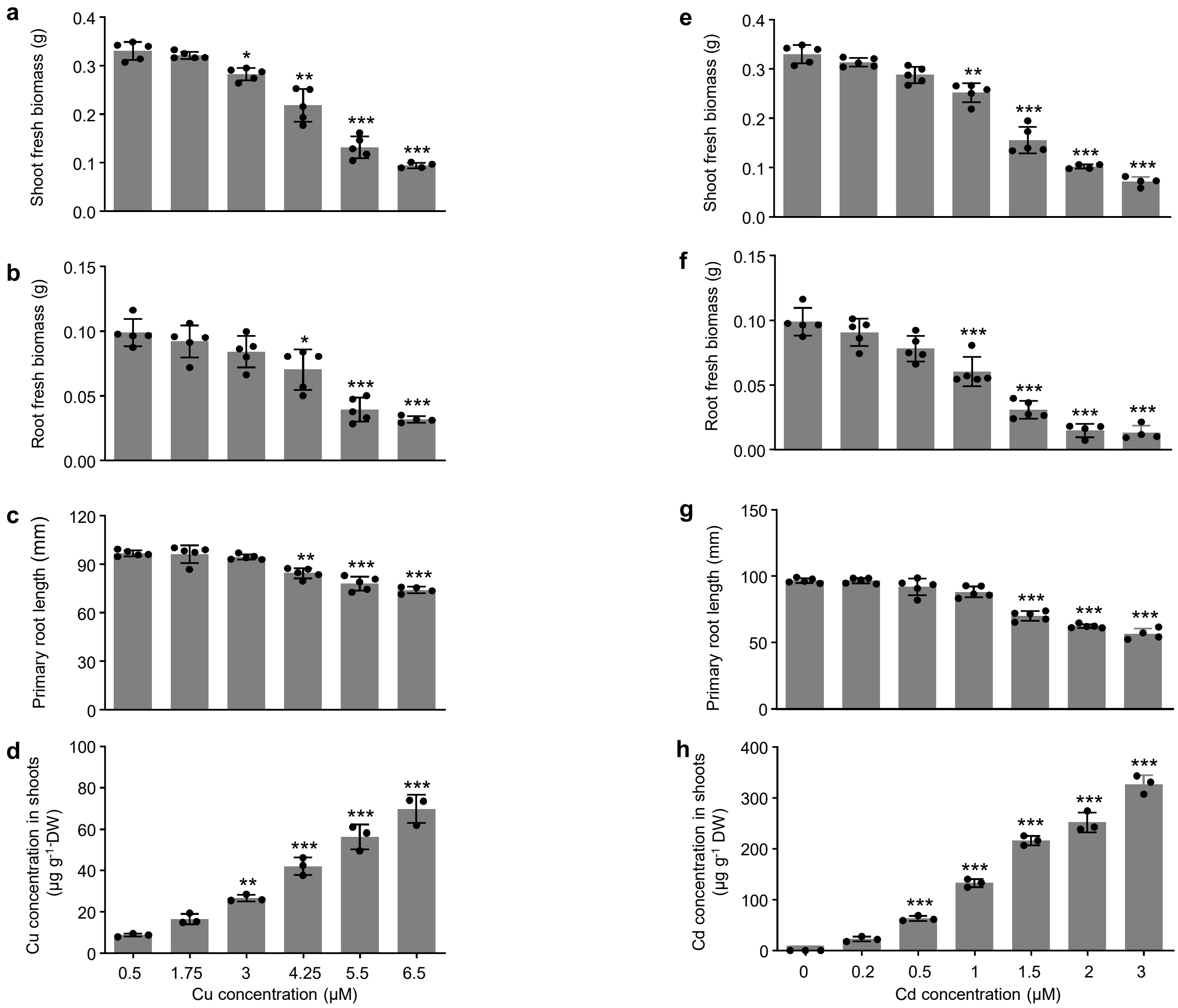

Supplementary Fig. 14 Data supporting that copper and cadmium toxicity correlate with accumulation of diphthamide-unmodified eEF2 protein. a-**h**, Shoot fresh biomass (**a**,**e**), root fresh biomass (**b**,**f**), primary root length (**c**,**g**), shoot Cu (**d**) and Cd (**h**) concentrations of Cu-exposed (**a**-**d**; shown in **Fig. 5c**) and Cd-exposed (**e**-**h**; shown in **Fig. 5d**) Arabidopsis wild-type seedlings. Shown are mean ± s.d., $n = 4$ (6.5 µM Cu, 2 µM Cd, 3 µM Cd) or $n = 5$ biologically independent pools (all other conditions) of tissues from 17 seedlings cultivated per petri plate (**a**,**b**,**e**,**f**), $n \geq 68$ (i.e., each seedling as specified for **a**,**b**,**e**,**f**) biologically independent seedlings (**c**,**g**), $n = 3$ biologically independent pools of 17 seedlings cultivated per petri plate (**d**,**h**). *, $P < 0.05$, **, $P < 0.01$, ***, $P < 0.001$, compared to the control treatment, based on one-way ANOVA with Games-Howell test (**a**,**e**), Scheffé test (**b**,**c**,**f**,**g**) or Tukey's test (**d**,**h**).

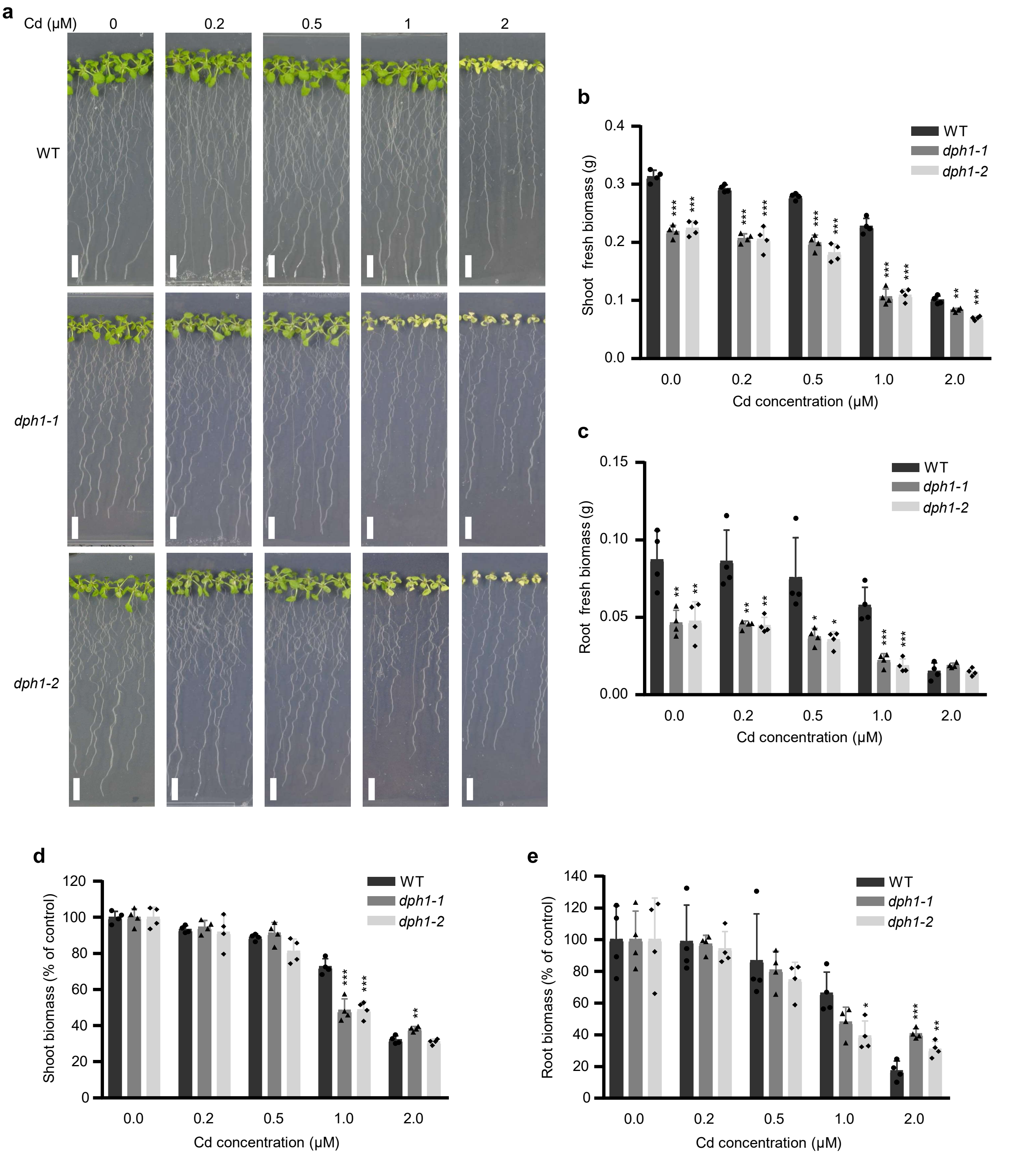

Supplementary Fig. 15 Sensitivity of *dph1* mutants to cadmium. a, Photographs of 18-d-old wild-type (WT), *dph1-1* and *dph1-2* seedlings grown in modified Hoagland medium supplemented with 0, 0.2, 0.5, 1, or 2 μM Cd. Scale bars, 10 mm. **b**-**c**, Shoot (**b**) and root (**c**) fresh biomass of the seedlings shown in (**a**). **d**-**e**, Shoot (**d**) and root (**e**) biomass as shown in (**b**) and (**c**), respectively, expressed relative to mean of the same genotype cultivated under the control (no Cd added) condition. Shown are mean ± s.d., $n = 4$ biologically independent samples, with each sample consisting of pooled tissues from 17 seedlings cultivated per petri plate; significant differences from WT: *, $P < 0.05$, **, $P < 0.01$ ***, $P < 0.001$, based on one-way ANOVA with Tukey's test (**b**-**e**).

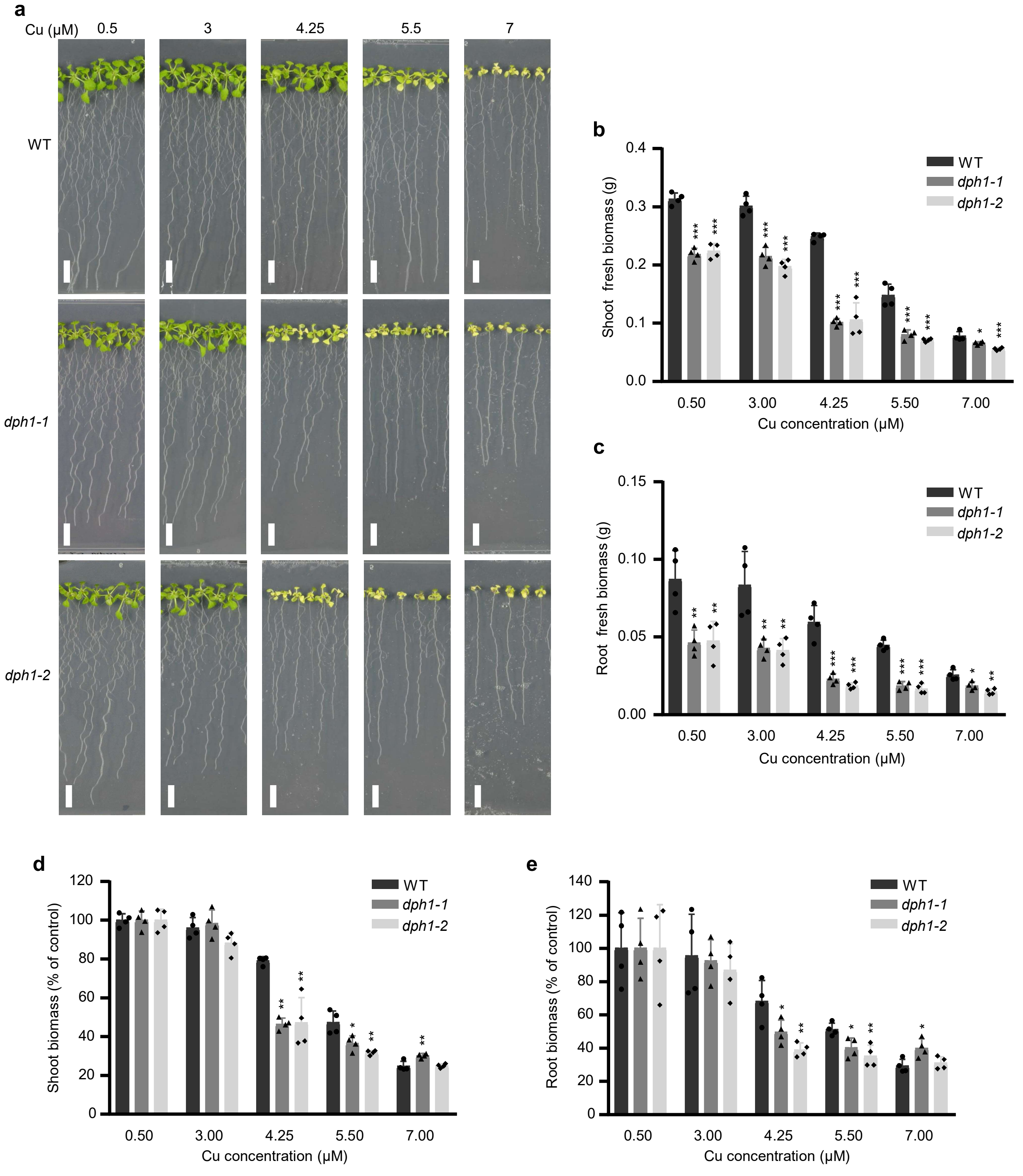

Supplementary Fig. 16 Sensitivity of *dph1* mutants to excess copper. a, Photographs of 18-d-old wild-type (WT), *dph1-1* and *dph1-2* seedlings grown in modified Hoagland medium supplemented with 0, 3, 4.25, 5.5 or 7 µM Cu. Scale bars, 10 mm. **b**-**c**, Shoot (**b**) and root (**c**) fresh biomass of the seedlings shown in (**a**). **d**-**e**, Shoot (**d**) and root (**e**) biomass as shown in (**b**) and (**c**), respectively, expressed relative to mean of the same genotype cultivated under the control (0.5 µM Cu) condition. Shown are mean ± s.d. (n = 4 replicate plates), with tissues from 17 seedlings pooled per replicate plate; significant differences from WT: *, $P < 0.05$, **, $P < 0.01$ ***, $P < 0.001$, based on one-way ANOVA with Tukey's test (**b**-**e**).

a

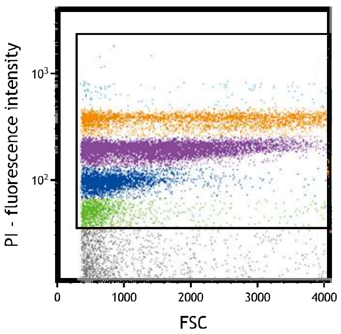

b

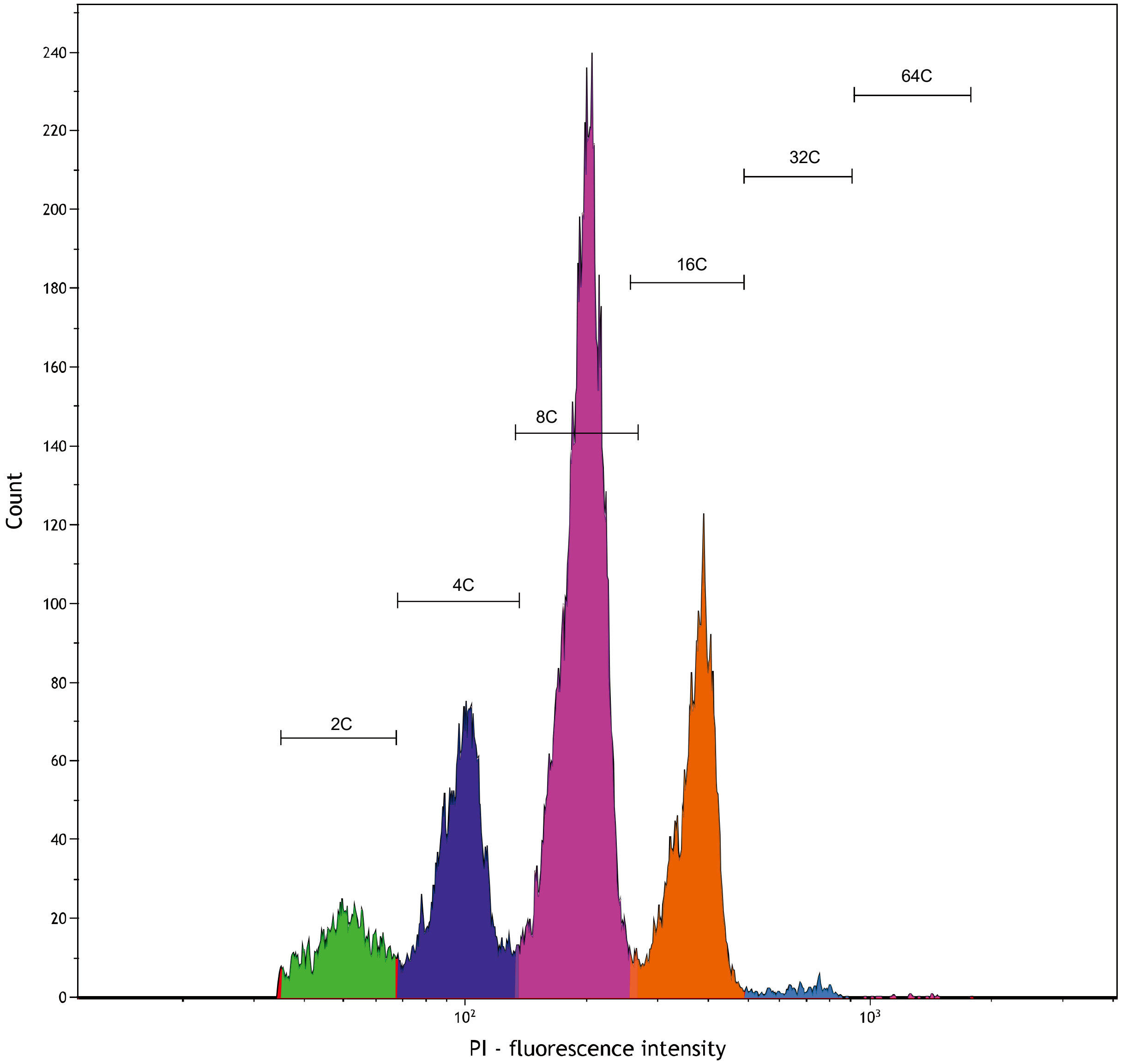

Supplementary Fig. 17 Gating strategy of flow cytometry. **a**, Histogram of propidium iodide (PI) fluorescence intensity and FSC (Forward Scattered light). Four high-intensity regions represent 2C (green), 4C (blue), 8C (violet), and 16C (orange), respectively. Nuclei were gated (marked by black rectangle) to exclude background signals. **b**, Histogram of gated nuclei with counts plotted *vs*. fluorescence intensity. Solid horizontal lines mark each ploidy range used for calculating the percentage of nuclei of different ploidy levels.

Supplementary Table 1. Relative abundance of diphthamide-modified peptide 684-GICFEVCDVVLHSDAIHR-701, a trypsin fragment of eEF2

**Genotype**	**Diphthamide-modified (%)**[a]
Wild type	96
dph1-1	< 0.5
dph1-2	< 1.0
Complemented line Y22-10	100

[a]Expressed relative to the sum of diphthamide-modified and non-modified peptide.

We quantified peak intensities at specific precursor *m/z* values (±3 ppm) for the two most abundant isotopes (non-modified: 532.5081, 532.7592; modified: 568.0359, 568.2865; z = +4) along chromatographic retention time.

Supplementary Table 2. Primers used in this study

Genotyping primers	Sequence (5' to 3')	Purpose
Dph1-1-LP	GCTTTCATCATCTTCGTCTGC	Genotyping *dph1-1* T-DNA mutants
Dph1-1-RP	CCACCGAGTCTTCAAACAATG	
Lbp745	AACGTCCGCAATGTGTTATTAAGTTGTC	
**RT-PCR and qRT-PCR primers**	**Sequence (5' to 3')**	**Purpose**
FqDPH1	TTTTGAGGCTGAGATTGCTCTA	RT-PCR and RT-qPCR of *DPH1*
RqDPH1	TTCTTTGTCCTCTTTGCAACAG	
FqACTIN8	TAAACTAAAGAGACATCGTTTCCA	RT-PCR of *ACTIN8*
RqACTIN8	TTTTTATCCGAGTTTGAAGAGGC	
FqUBQ10	GGCCTTGTATAATCCCTGATGAATAAG	RT-qPCR of *UBQ10*
RqUBQ10	AAAGAGATAACAGGAACGGAAACATAGT	
**Cloning primers**	**Sequence (5' to 3')**	**Purpose**
Green-P-DPH1-F	AACAGGTCTCAACCTACAGCTTTTTAAGACAAGCAGAGC	Amplify *DPH1* promoter for GreenGate cloning
Green-P-DPH1-R	AACAGGTCTCATGTTACTTTGGCCTGAAACATAAGAG	
Green-CDS-DPH1-F	AACAGGTCTCAGGCTATGGAGCTTTCTGATCCAAACA	Amplify *DPH1* coding region for GreenGate cloning
Green-CDS-DPH1-R	AACAGGTCTCACTGAAACTACAGAAGAAGGTAAAGGGTTTC	
Green-Termi-DPH1-F	AACAGGTCTCACTGCAATGCTGACAGAGTAAATTCAAC	Amplify *DPH1* terminator for GreenGate cloning
Green-Termi-DPH1-R	AACAGGTCTCATAGTAAAATCAGCTGAAACTTATTCTCT	
pYDL-F	ACGCCTCGAGATGACTTCGAAAGTTTATGATCC	Cloning renilla and firefly coding region together with polylinker region into pMatrix
pYDL-R	ACGACTAGTTTACAATTTGGACTTTCCGCC	
