# Supplementary material for: Translational fidelity and growth of Arabidopsis require stress-sensitive diphthamide biosynthesis
Source: Nat Commun. 2022 Jul 11;13:4009. doi: 10.1038/s41467-022-31712-7 (PMC9273596; doi:10.1038/s41467-022-31712-7)
Supplement: Supplementary file 3 — Description of Additional Supplementary Files [file 41467_2022_31712_MOESM3_ESM.pdf]

Description of Additional Supplementary Files

File Name: Supplementary Data 1
Description: **Comparative transcriptomics of wild-type and *dph1* mutant Arabidopsis leaves.** Transcripts per kilobase million (TPM) for all genes of which transcripts were detected, genes of which transcripts differed in abundance from the wild type in the *dph1-1* and *dph1-2* mutant, respectively, as well as genes of which transcript levels were different between the wild type and both the *dph1* mutants.

File Name: Supplementary Data 2
Description: **Gene Ontology (GO) term enrichment of transcripts differentially abundant between both *dph1* mutants and wild type.**

File Name: Supplementary Data 3
Description: **Ribosomal protein- and protease-encoding genes of which transcript levels differed between the wild type and both *dph1* mutants.**
