# Supplementary material for: Translational fidelity and growth of Arabidopsis require stress-sensitive diphthamide biosynthesis
Source: Nat Commun. 2022 Jul 11;13:4009. doi: 10.1038/s41467-022-31712-7 (PMC9273596; doi:10.1038/s41467-022-31712-7)
Supplement: Supplementary file 7 — Reporting Summary [file 41467_2022_31712_MOESM7_ESM.pdf]

nature research

Corresponding author(s): Kraemer, Ute

Last updated by author(s): Feb 28, 2022

Reporting Summary

Nature Research wishes to improve the reproducibility of the work that we publish. This form provides structure for consistency and transparency in reporting. For further information on Nature Research policies, see our Editorial Policies and the Editorial Policy Checklist.

Statistics

For all statistical analyses, confirm that the following items are present in the figure legend, table legend, main text, or Methods section.

n/a	Confirmed	
☐	☒	The exact sample size (n) for each experimental group/condition, given as a discrete number and unit of measurement
☐	☒	A statement on whether measurements were taken from distinct samples or whether the same sample was measured repeatedly
☐	☒	The statistical test(s) used AND whether they are one- or two-sided<br>*Only common tests should be described solely by name; describe more complex techniques in the Methods section.*
☒	☐	A description of all covariates tested
☐	☒	A description of any assumptions or corrections, such as tests of normality and adjustment for multiple comparisons
☐	☒	A full description of the statistical parameters including central tendency (e.g. means) or other basic estimates (e.g. regression coefficient) AND variation (e.g. standard deviation) or associated estimates of uncertainty (e.g. confidence intervals)
☐	☒	For null hypothesis testing, the test statistic (e.g. F, t, r) with confidence intervals, effect sizes, degrees of freedom and P value noted<br>*Give P values as exact values whenever suitable.*
☒	☐	For Bayesian analysis, information on the choice of priors and Markov chain Monte Carlo settings
☒	☐	For hierarchical and complex designs, identification of the appropriate level for tests and full reporting of outcomes
☒	☐	Estimates of effect sizes (e.g. Cohen's d, Pearson's r), indicating how they were calculated

Our web collection on statistics for biologists contains articles on many of the points above.

Software and code

Policy information about availability of computer code

Data collection	LightCycler480 Software 1.5 was used for RT-qPCR.<br>LAS_X_3.0.11 was used to capture confocal images.<br>Olympus OlyVIA was used to capture fluorescent images.<br>ZEN2 was used to capture images under DIC mode.<br>Fusion FX7 was used to image the blots.<br>Gen5_2.07 was used to measure luciferase activities.<br>Partec FloMax was used for flow cytometry assay.<br>iTEVA 9.8 was used for ICP analysis.<br>Thermo orbitrap fusion mass spectrometer was used for mass spectrometry.<br>CCM-200 plus chlorophyll content meter was used for chlorophyll measurement.
Data analysis	Leaf areas, meristem length, cell sizes, cell numbers, intensity of western blot bands, and fluorescence intensity were determined through ImageJ 1.52a. MEGA6.0 was used to build neighboring-joining tree. Thermo Proteome Discoverer 2.4 was used to identify target peptides in mass spectrometry. SPSS 19 was used for all the statistical analysis. GraphPad Prism 9 and Microsoft Excel 2010 were used to make graphs. Microsoft PowerPoint 2010 was used to assemble figures.

For manuscripts utilizing custom algorithms or software that are central to the research but not yet described in published literature, software must be made available to editors and reviewers. We strongly encourage code deposition in a community repository (e.g. GitHub). See the Nature Research guidelines for submitting code & software for further information.

Data

Policy information about availability of data

All manuscripts must include a data availability statement. This statement should provide the following information, where applicable:

- Accession codes, unique identifiers, or web links for publicly available datasets
- A list of figures that have associated raw data
- A description of any restrictions on data availability

All data are available in the main text or the supplementary materials.

Field-specific reporting

Please select the one below that is the best fit for your research. If you are not sure, read the appropriate sections before making your selection.

☒ Life sciences ☐ Behavioural & social sciences ☐ Ecological, evolutionary & environmental sciences

For a reference copy of the document with all sections, see nature.com/documents/nr-reporting-summary-flat.pdf

Life sciences study design

All studies must disclose on these points even when the disclosure is negative.

Sample size	The sample size used was based on our experience and the literature. We think the sample size we used is large enough to get representative results. The sample size has been shown in each figure legend.
Data exclusions	No data were excluded.
Replication	Numbers of within-experiment replicates and their precise nature are provided in Figure legends. All the experiments were repeated independently at least twice observing the same fundamental results (statistical significance and direction of differences, e.g. in mutant lines vs. wild type).
Randomization	Plants or plates with seedlings were put randomly in the phytochamber and their position were changed regularly.
Blinding	Experiments were not blinded. Data were collected according to different genotype of plants.

Reporting for specific materials, systems and methods

We require information from authors about some types of materials, experimental systems and methods used in many studies. Here, indicate whether each material, system or method listed is relevant to your study. If you are not sure if a list item applies to your research, read the appropriate section before selecting a response.

Materials & experimental systems

n/a	Involved in the study	
☐	☒	Antibodies
☐	☒	Eukaryotic cell lines
☐	☐	Palaeontology and archaeology
☒	☐	Animals and other organisms
☒	☐	Human research participants
☒	☐	Clinical data
☒	☐	Dual use research of concern

Methods

n/a	Involved in the study	
☒	☐	ChIP-seq
☐	☒	Flow cytometry
☒	☐	MRI-based neuroimaging

Antibodies

Antibodies used	All antibody information is provided in the Methods section.
Validation	3C2 and 10G8 antibodies (1., 2.) and TCTP1 antibodies (3.) were verified in the publications listed below. All other antibodies were ordered from companies and validated by the suppliers and other previous researchers.<br>1. Hawer, H. et al. Importance of diphthamide modified EF2 for translational accuracy and competitive cell growth in yeast. PLoS One 13, e0205870, doi:10.1371/journal.pone.0205870 (2018).<br>2. Stahl, S. et al. Loss of diphthamide pre-activates NF-κB and death receptor pathways and renders MCF7 cells hypersensitive to tumor necrosis factor. Proceedings of the National Academy of Sciences 112, 10732-10737 (2015).<br>3. Brioudes, F. et al. Translationally controlled tumour protein is a conserved mitotic growth integrator in animals and plants. PNAS 107, 16384-9, doi:10.1073/pnas.1007926107 (2010).

Eukaryotic cell lines

Policy information about cell lines

Cell line source(s)	MCF-7
Authentication	ATCC HTB-22
Mycoplasma contamination	tested, confirmed negative
Commonly misidentified lines (See ICLAC register)	*Name any commonly misidentified cell lines used in the study and provide a rationale for their use.*

Palaeontology and Archaeology

Specimen provenance	*Provide provenance information for specimens and describe permits that were obtained for the work (including the name of the issuing authority, the date of issue, and any identifying information).*
Specimen deposition	*Indicate where the specimens have been deposited to permit free access by other researchers.*
Dating methods	*If new dates are provided, describe how they were obtained (e.g. collection, storage, sample pretreatment and measurement), where they were obtained (i.e. lab name), the calibration program and the protocol for quality assurance OR state that no new dates are provided.*

☐ Tick this box to confirm that the raw and calibrated dates are available in the paper or in Supplementary Information.

Ethics oversight	*Identify the organization(s) that approved or provided guidance on the study protocol, OR state that no ethical approval or guidance was required and explain why not.*

Note that full information on the approval of the study protocol must also be provided in the manuscript.

Flow Cytometry

Plots

Confirm that:

☒ The axis labels state the marker and fluorochrome used (e.g. CD4-FITC).

☒ The axis scales are clearly visible. Include numbers along axes only for bottom left plot of group (a 'group' is an analysis of identical markers).

☐ All plots are contour plots with outliers or pseudocolor plots.

☒ A numerical value for number of cells or percentage (with statistics) is provided.

Methodology

Sample preparation	The information is given in the Methods section.
Instrument	CyFlow SL 3-Colour FCM System (Sysmec Partec, Münster, Germany)
Software	FloMax was used to collect and analyze the flow cytometry data.
Cell population abundance	20,000 nuclei were analyzed each sample. The samples were filtered through a 30 µm cell strainer to remove debris. The purity of samples was further checked by fluorescent microscopy to make sure only nuclei were present in the samples.
Gating strategy	Threshold was set to exclude remaining debris. Histogram of FL2 (PI-fluorescent intensity) was used to identify fluorescence intensities corresponding to the ploidy of nuclei (2C, 4C, 8C, 16C, and 32C). Regions were set to determine the number of nuclei.

☒ Tick this box to confirm that a figure exemplifying the gating strategy is provided in the Supplementary Information.
